# Readiness of health facilities to provide safe childbirth in Liberia: a cross-sectional analysis of population surveys, facility censuses and facility birth records

**DOI:** 10.1186/s12884-022-05301-x

**Published:** 2022-12-20

**Authors:** Jessica King, Alfred K. Tarway-Twalla, Mardieh Dennis, Musu Pusah Twalla, Patrick K. Konwloh, Chea Sanford Wesseh, Bentoe Zoogley Tehoungue, Geetor S. Saydee, Oona Campbell, Carine Ronsmans

**Affiliations:** 1 London School of Hygiene and Tropical Medicine, Keppel St., London, WC1E 7HT UK; 2 University of Liberia, Capitol Hill, 1000 Monrovia, Liberia; 3 Last Mile Health, Tubman Boulevard, Monrovia, Liberia; 4 Ministry of Health, P.O.Box 9009, 1000 Monrovia, Liberia

**Keywords:** Facility delivery, Volume of births, Signal functions, Emergency obstetric and neonatal care

## Abstract

**Background:**

The provision of quality obstetric care in health facilities is central to reducing maternal mortality, but simply increasing childbirth in facilities not enough, with evidence that many facilities in sub-Saharan Africa do not fulfil even basic requirements for safe childbirth care. There is ongoing debate on whether to recommend a policy of birth in hospitals, where staffing and capacity may be better, over lower level facilities, which are closer to women’s homes and more accessible. Little is known about the quality of childbirth care in Liberia, where facility births have increased in recent decades, but maternal mortality remains among the highest in the world. We will analyse quality in terms of readiness for emergency care and referral, staffing, and volume of births.

**Methods:**

We assessed the readiness of the Liberian health system to provide safe care during childbirth use using three data sources: Demographic and Health Surveys (DHS), Service Availability and Readiness Assessments (SARA), and the Health Management Information System (HMIS). We estimated trends in the percentage of births by location and population caesarean-section coverage from 3 DHS surveys (2007, 2013 and 2019–20). We examined readiness for safe childbirth care among all Liberian health facilities by analysing reported emergency obstetric and neonatal care signal functions (EmONC) and staffing from SARA 2018, and linking with volume of births reported in HMIS 2019.

**Results:**

The percentage of births in facilities increased from 37 to 80% between 2004 and 2017, while the caesarean section rate increased from 3.3 to 5.0%. 18% of facilities could carry out basic EmONC signal functions, and 8% could provide blood transfusion and caesarean section. Overall, 63% of facility births were in places without full basic emergency readiness. 60% of facilities could not make emergency referrals, and 54% had fewer than one birth every two days.

**Conclusions:**

The increase in proportions of facility births over time occurred because women gave birth in lower-level facilities. However, most facilities are very low volume, and cannot provide safe EmONC, even at the basic level. This presents the health system with a serious challenge for assuring safe, good-quality childbirth services.

**Supplementary Information:**

The online version contains supplementary material available at 10.1186/s12884-022-05301-x.

## Background

The Millennium Development Goal era saw maternal mortality decline across sub-Saharan Africa (SSA) [1, 2], although few countries met the target of reducing the maternal mortality ratio (MMR) by three-quarters between 1990 and 2013 [3]. The Sustainable Development Goals have set an even more ambitious goal of reducing the global MMR to below 70 deaths per 100 000 live births by 2030, requiring a substantial scaling-up of effective strategies to reduce maternal mortality, particularly in SSA [4, 5].

The provision of quality obstetric care in health facilities is central to reducing maternal mortality [6–8]. Coverage of facility births has increased dramatically, including in SSA, where births have mostly increased in low-level, low-volume health facilities close to where women live [9, 10] Simply increasing childbirth in facilities is not enough to reduce maternal mortality [11–13]. Many facilities in SSA do not fulfil the basic requirements for safe emergency obstetric and neonatal care (EmONC) [9, 14], staffing is often inadequate [14] and the capacity for emergency referral may be poor [15, 16]. This is compounded in lower level facilities, where the volume of births is low and staff lack the knowledge and skills to provide safe childbirth [9]. Analysis of maternal deaths has shown that proximity to a lower level health facility does not protect against direct maternal mortality, and only proximity to a hospital reduces a woman’s likelihood of dying during childbirth [17]. The poor quality of obstetric care in low level facilities has led some to recommend a policy of birth in hospital rather than in dispensaries or clinics [10], though there is no global consensus on where women should give birth [18].

Little is known about the quality of obstetric care in Liberia. The proportion of births in health facilities was 80% in 2019–20 [19], but the level or sector where these took place was not reported. Only 5.3% of births were by caesarean section, suggesting poor access to life-saving surgical care [19]. A survey of 71 facilities with maternity waiting homes found that facilities had a mean readiness of 4.3 out of seven BEmONC signal functions [20], and a survey of surgical capacity in 35 hospitals identified they could carry out a median of three of seven essential obstetric and gynaecological procedures [21], suggesting quality of care is poor even in high level facilities.

The objectives of our paper are to describe trends over time in location of childbirth and in caesarean section rates in rural and urban areas in Liberia; and to describe the readiness of facilities, by level and sector, to provide safe childbirth conditions, including signal functions for EmONC, staffing, and volume of births and caesarean sections [22].

## Methods

### Setting

Liberia had a population of approximately five million in 2021 and a total fertility rate of 4.1 children per woman [23]. The Liberian health system, weakened by civil war, was further damaged by the 2014–16 Ebola epidemic [24]. Maternal mortality remains high, at an estimated 661 per 100,000 live births in 2017, a decrease of 26% from 2000 [5]. The estimated neonatal mortality in 2020 was 31 per 1000 live births, a decrease of 35% from 2000 [25].

The three levels of facility which provide childbirth care in Liberia are clinics, health centres and hospital. Clinics, the lowest level of facility, are expected to provide routine antenatal, labour, and postnatal care, and deal with certain obstetric emergencies, and should be staffed by one nurse and one midwife. Health centres are expected to provide BEmONC and should be staffed with at least two physician assistants, four midwives and one nurse. Finally, hospitals (the highest level) are intended to provide Comprehensive Emergency Obstetric and Neonatal Care (CEmONC), including caesarean section and blood transfusion, and be staffed a doctor, three physician assistants, six midwives and ten nurses.

### Data sources

We used three data sources: the DHS surveys (2007, 2013 and 2019–20), the Liberia Service Availability and Readiness Assessment (SARA) (2018) and the Liberia Health Management Information System (HMIS) 2019.

The DHS surveys were used to estimate coverage of facility births by type of facility and caesarean section rates among all live births in the 5 years preceding the survey. The total sample size was 19,106 live births across the three surveys. To assess staffing and EmONC signal functions, we used data from the SARA 2018 which included all health facilities in Liberia in December 2017 and January 2018. We removed duplicate assessments and those with incomplete data on childbirth services, and included facilities in our analysis only if they reported offering childbirth services. To examine volume of births and caesarean sections, we used data from facilities reporting at least one live birth in 2019 to the Health Management Information System (HMIS). To enable us to jointly assess volume of births and signal functions, we matched facilities in the HMIS and SARA datasets on name, region and district.

### Analysis

We estimated the percentage of births in public hospitals, public health centres, public clinics/other public, private facilities, home, and other/missing for each DHS, using the mid-point of the 5-year recall period for each survey (2004 for 2007, 2010 for 2013 & 2017 for 2019–2020). Data on private facilities by level were not available in the DHS. Full details of the birth location indicator definitions are given in the Additional file 1: Table S1. We also report the percentage of births by caesarean section for each DHS, examining each singleton birth and for the neonate who was born last in each multiple birth.

Readiness to deliver EmONC was assessed through the availability of signal functions [22]. We defined facilities as having readiness to provide BEmONC-1 if they reported having carried out six of the seven signal interventions for management of basic obstetric emergencies at least once in the 12 months preceding the 2018 SARA visit, and if the necessary drugs or equipment were observed in the facility. The six signal functions were parenteral administration of antibiotics, parenteral administration of oxytocin, parenteral administration of anticonvulsants, manual removal of placenta, removal of retained products and neonatal resuscitation (full details Additional file 1: Table S2). We removed the requirement for assisted vaginal delivery as part of BEmONC because this is so rarely provided. Facilities were defined as having CEmONC-1 readiness if they could provide the six basic signal functions and the two comprehensive functions, caesarean section and blood transfusion.

Readiness to make an emergency referral was assessed through availability of vehicles and telephones reported in SARA 2018. A facility was defined to have readiness for emergency referral if it had either a) a functional ambulance or other vehicle stationed at the facility, or b) access to an ambulance or other vehicle stationed at another facility, and a functioning telephone (landline or mobile) supported by the facility.

Skilled birth attendants (SBAs) were defined as doctors, physician assistants, midwives and nurses. Total numbers of SBAs, and numbers by cadre, were calculated from the numbers of each cadre reported working at the facility in the 2018 SARA, irrespective of their full- or part-time status. Physician assistants were grouped with doctors for analyses by cadre due to low numbers.

The number of live births per facility reported to HMIS in 2019 were grouped in five categories, as suggested by Kruk et al. [9]: < 53 a year (or < 1 per week), 53–183 a year (one every two days), 184–365 a year (up to one a day), 366–500 a year, and > 500 a year [9]. Five hundred births a year is an internationally used threshold, for example in the UK and the US [26, 27]. Facilities which reported at least one caesarean section were grouped into four volume categories for caesareans: < 25 a year, 25–99 a year, 100–199 a year and ≥ 200 a year. We calculated the percentage of facility births and caesarean sections occurring in facilities with different levels of EmONC or volume categories directly, by summing the number of live births and caesarean sections reported by each facility in the HMIS 2019 for each EmONC readiness and volume category.

All analysis was carried out in Stata version 17.0.

## Results

### National trends in percentage of births at facilities over time

**Fig. 1:**
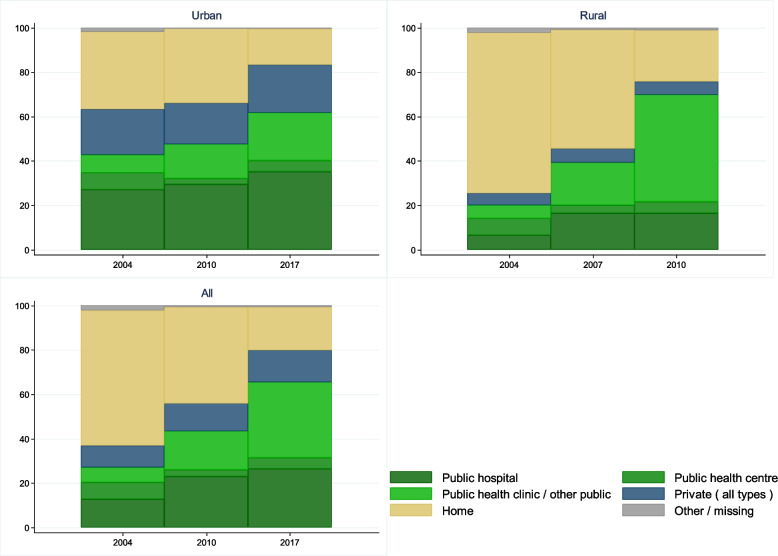
Trends in place of birth by urban and rural residence (all live births in 5-year recall period, DHS 2007, 2013 & 2019–20; the midpoint shown is the calendar year of the halfway point of the recall period for each survey)

The percentage of births in health facilities more than doubled from 37% in 2004 to 80% in 2017 (Fig. 1 and Additional file 1: Table S3). By 2017, the gap in coverage of facility births between urban and rural areas nearly disappeared. The biggest rise in facility births was in public health clinics, particularly in rural areas where they rose from 6 to 48% of all births. There was also an increase in hospital births, from 13 to 26% of all births, and in private facilities, from 10 to 14% of births between 2004 and 2017. Very few (5%) women gave birth in health centres.

### Emergency obstetric and neonatal care signal functions

**Fig. 2:**
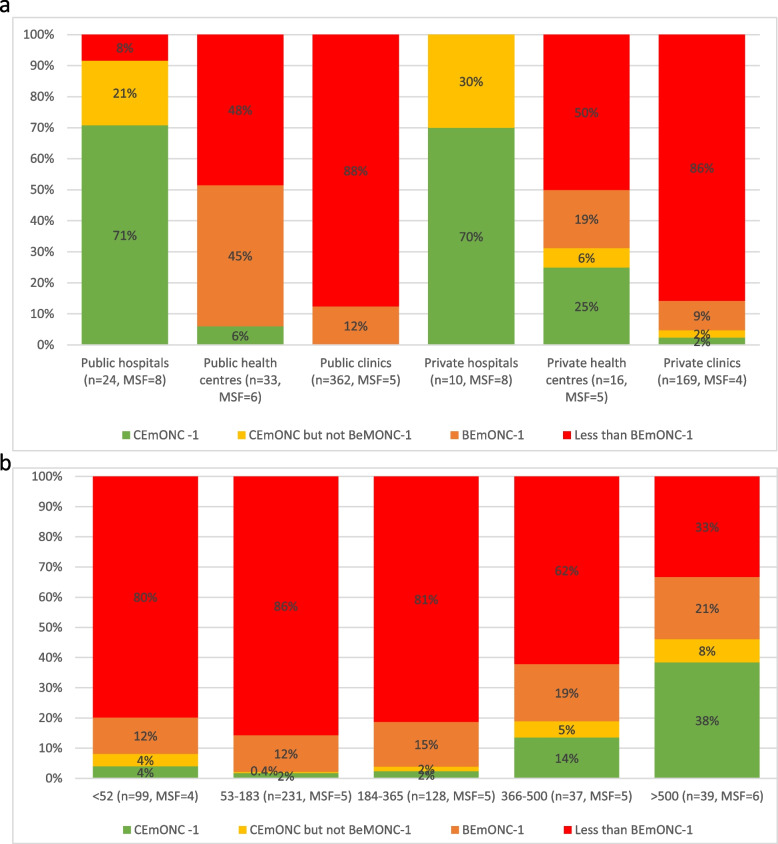
**a** Emergency obstetric and neonatal care signal functions, by facility level and ownership (SARA 2018). BEmONC = basic emergency obstetric and neonatal care, CEmONC = comprehensive emergency obstetric and neonatal care, MSF = median signal functions. CEmONC but not BeMONC-1 indicates readiness for blood transfusion and caesarean section, but not all BeMONC-1 signal functions. **b** Emergency obstetric and neonatal care signal functions, by yearly volume of births (SARA 2018 and HMIS 2019). BEmONC = basic emergency obstetric and neonatal care, CEmONC = comprehensive emergency obstetric and neonatal care, MSF = median signal functions. CEmONC but not BeMONC-1 indicates readiness for blood transfusion and caesarean section, but not all six BeMONC-1 signal functions

On the 765 entries in the 2018 SARA dataset, 727 were non-duplicate assessments with complete data on childbirth services, of which 614 facilities reported offering childbirth services. Among these 614 facilities, there were 362 public clinics (59%), 33 public health centres (5%), 24 public hospitals (4%), 169 private clinics (28%), 16 private health centres (3%) and 10 private hospitals (2%). Only a small proportion (18%) of facilities had the readiness to carry out all six BEmONC-1 functions (Fig. 2a and Additional file 1: Table S5). This was highest among public hospitals (71%), and lowest among public and private clinics (12%). Facilities which did not have BEmONC-1 readiness could carry out a median of five signal functions. Readiness was highest for parenteral antibiotics, which were available in 100% of hospitals and health centres, and 96% of clinics (Additional file 1: Table S5). The lowest coverage was for removal of retained products (76% of hospitals, 57% of health centres and 27% of clinics). Assisted vaginal delivery, excluded from the BEmONC-1 indicator, could only be carried out in 14% of facilities.

There were 34 facilities with full CEmONC-1 readiness (6%), though an additional 13 had the readiness to carry out caesarean section and blood transfusion, without full BEmONC-1 readiness (Fig. 2a and Additional file 1: Table S5). Nearly all (92%) public hospitals and 6% of public health centres had the readiness to carry out the two CEmONC functions (regardless of BEmONC-1 readiness), compared to 100% of private hospitals, 31% of private health centres and 5% of private clinics. Only 42% of facilities had the readiness to make an emergency referral (defined as having an ambulance onsite, or an ambulance offsite and a telephone), and this was just 33% in public clinics (Additional file 1: Table S5).

### Staffing

Nearly all health facilities (99.7%) reported employing at least one skilled birth attendant (SBA, Additional file 1: Table S7). All but two public clinics had an SBA, though 29% did not employ any midwife. Both public and private hospitals had reasonable numbers of midwives (median 11 and 7 respectively), exceeding the median number of doctors.

### Volume of births

Five hundred eighty-six facilities reported at least one live birth in 2019 to the Health Management Information System (HMIS), of which 534 were matched on name and location to the SARA datset. Among these 534 facilities, the median number of live births was 140 a year, or around 12 a month (Fig. S1). Public and private clinics had the lowest volumes, with 63 and 84% respectively reporting fewer than 183 births per year. While public hospitals had the highest volumes, 4 (17%) had fewer than 183 a year.

Among the 458 (86%) facilities with fewer than 365 births a year, 83% did not have readiness to deliver the six BEmONC-1 functions (Fig. 2b), and only 40% were ready to make an emergency referral (Additional file 1: Table S6). Even among the 37 facilities with between 366 and 500 births a year, 68% were not able to provide all six BEmONC-1 functions. Readiness is considerably improved among the facilities with over 500 births year, of which 59% could carry out the six BEmONC-1 functions, and 46% the two CEmONC functions.

### Coverage of caesarean section

**Fig. 3:**
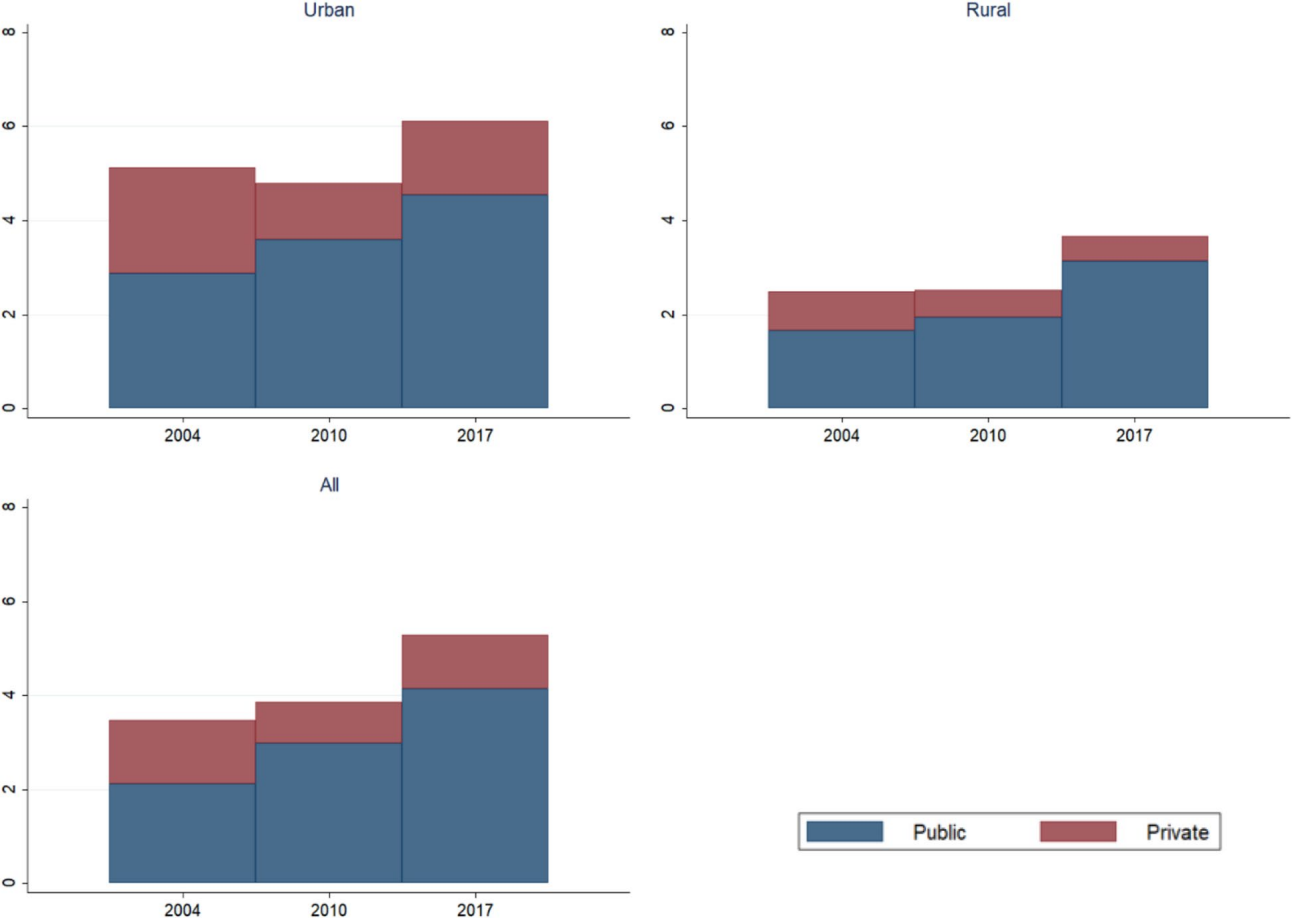
Population based percentage of births via caesarean section by urban rural residence and sector (all live singleton births and last born neonate for multiple births in 5-year recall period; DHS 2007, 2013 & 2019–20; the midpoint shown is the calendar year of the halfway point of the recall period for each survey)

The proportion of babies born by caesarean section increased from 3.3% in 2004 to 5.0% in 2017 (Fig. 3). 6.1% of babies were born by caesarean by in urban areas in 2017 compared to 3.7% in rural areas.

### Volume, signal functions and caesarean sections by number of births and number of facilities

**Fig. 4:**
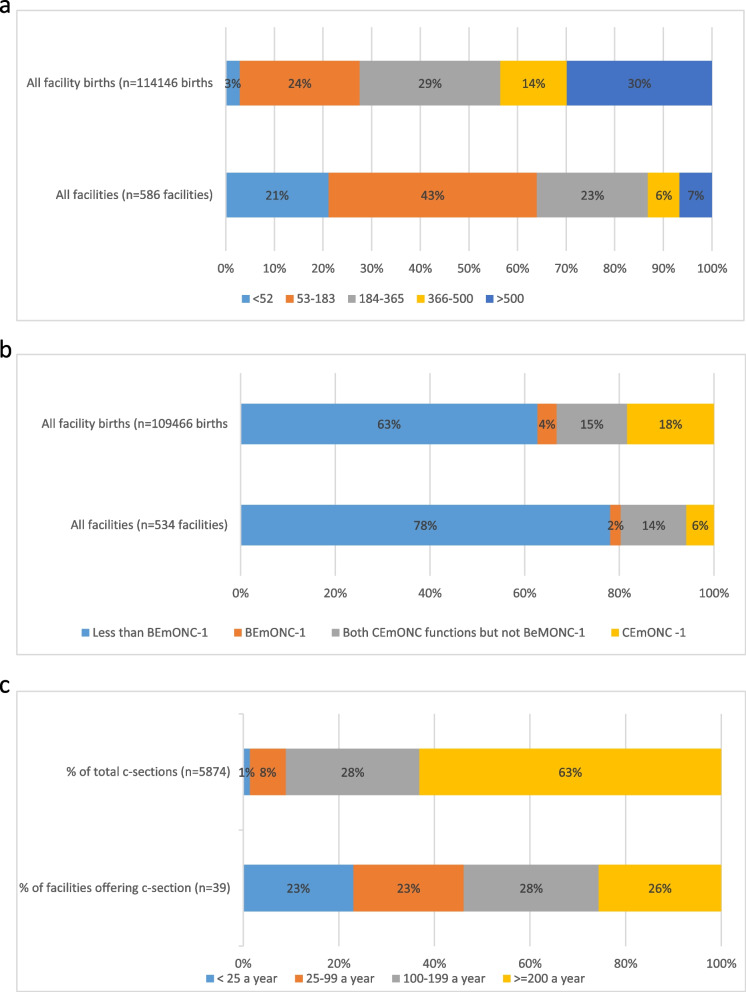
**a** Facility births, and number of facilities, by yearly volume of live births (HMIS 2019). **b** Facility births, and number of facilities, by EmONC readiness (SARA 2018 and HMIS 2019). **c** Caesarean sections, and number of facilities, by yearly volume of caesarean sections (HMIS 2019)

Figure 4a-c presents the distribution of the number of births and the number of facilities in 2019 by volume of births and readiness to provide EmONC. The 316 (54%) facilities with fewer than 183 births a year represented only 27% of all facility births in Liberia (Fig. 4a). Worryingly, 78% of live births took place in facilities which did not have BEmONC-1 readiness, representing 92% of all facilities offering childbirth care (Fig. 4b).

The same pattern can be observed when analysing caesarean section births (Fig. 4c). 46% of facilities offering caesarean section did fewer than 100 per year, yet they only accounted for 9% of all caesarean sections in Liberia. 63% of all caesarean sections were in the 10 facilities which reported at least 200 caesarean sections a year.

## Discussion

The percentage of births in health facilities increased greatly in Liberia between 2004 and 2017, from 37 to 80%. Most of this increase occurred in rural areas, thereby closing the gap between urban and rural areas. Caesarean section rates, on the other hand, remained low. By 2017, caesarean section rates were 3.7% in rural areas and 6.1% in urban areas, well below the 9–19% population coverage associated with decreases in maternal and neonatal mortality [28, 29], and the 10% threshold recommended by the WHO [30]. However, this is not unusually low for the region, with an estimated caesarean section rate of 5.0% across SSA [31]. The increase in facility births, equivalent to an annual growth rate of 6.1%, was faster than the increase in most other countries in SSA in the same period, which had a median growth rate of 3.2% in facility births [32].

Most of the increase in facility births was achieved through greater use of public clinics, the lowest level public health facility in Liberia. By 2017, a third (34%) of all births took place in public health clinics, increasing to nearly half (48%) in rural areas. Very few births took place in private facilities or in health centres. This increase in use of facilities may be the result of a number of government policies. User fees in all public health facilities were suspended in 2006 [33], and the government implemented a Basic Package of Health Services in 2009, which included expanding provision of maternal and neonatal health services at primary and secondary level facilities [34]. There has also been a focus on rapidly expanding the health workforce, with the number of midwives increasing by 28% between 2006 and 2010 [35].

Analysis of signal function readiness and volume of births suggest that only 45 (12%) of 362 public clinics could offer all six BEmONC-1 signal functions and 222 (63%) had fewer than 183 births per year. Overall, the 316 (54%) facilities doing fewer than 183 births per year in Liberia accounted for only 27% of all facility births. Volume of births and EmONC functions were closely related, and coverage of all six BEmONC signal functions was only reasonable in facilities doing more than 500 births per year. Even so, 16 (41%) of these large facilities did not achieve full BEmONC-1 readiness. This is despite the fact that facilities were defined as ready to perform a function if it had been carried out in the last 12 months, as opposed to the WHO’s more stringent requirement of the last three months. We also excluded assisted delivery from our BEmONC definition; levels of readiness would have been even lower if included.

Staffing at higher level facilities generally met the requirements of the Liberia Ministry of Health: hospitals are required to be staffed by four doctors and physician assistants and six midwives; the 7 and 11 respectively observed in public hospitals far exceeds this number. In public health centres, a median of one doctor or physician assistant and six midwives are employed, compared to the required numbers of two and four. Public health clinics employed a median of one midwife, as required by the Ministry of Health, though 29% did not have a single midwife, with a nurse as the only skilled birth attendant.

The increase in facility childbirth in Liberia is remarkable, particularly over a period of massive challenges to the population and the health system, including an Ebola epidemic which eroded trust in the government and health system [36], killed an estimated 7% of all healthcare workers [37], and decreased uptake of childbirth in health facilities [38–41].

The vast majority of facility births are in low volume facilities without the full set of BEmONC signal functions. This presents the Ministry of Health with the challenge of weighing the costs and feasibility of improving the very large number of small, low quality facilities with expanding access to higher volume, higher quality facilities and encouraging women to give birth further from home. The latter could create concerns for geographical equity: 48% of rural women give birth in public clinics, so doing so could re-open the urban-rural coverage gap. One option is the expansion of maternity waiting homes, which are already in use in Liberia, though often attached to poor quality facilities [20]. However, there is limited evidence that waiting homes have reduced maternal mortality elsewhere [42]. Another approach is to improve mechanisms for emergency referral from low to high level facilities if a woman develops complications during labour, though the evidence base for successful interventions is weak [43]. Surprisingly, health centres are not important as a place for childbirth in Liberia, which contrasts with other countries in SSA where the referral pathway to hospital often transitions via better equipped health centres, or other intermediate level facilities [14]. This suggests that interventions to improve emergency referrals (including investment in telephones and vehicles, as well as training and support of healthcare professionals) will have to be put in place in a large number of small and potentially under-resourced clinics. Investments in non-health specific infrastructure, such as roads, are also likely to have a significant impact on access to emergency care.

Facilities were generally well staffed, with most meeting the minimum requirements set out by the Ministry of Health. While this is a clear strength in the Liberian health system, low service volumes mean that human resources are used inefficiently. The WHO benchmark is that one midwife is required per 175 births annually [44]. The median public clinic in Liberia employed one midwife, as required by Ministry of Health minimum standards, but reported only 144 births a year, suggesting that volumes at individual facilities could increase without requiring additional staffing. This also aligns with a workforce availability analysis which found that Liberia had 97% of the midwives required to serve the population, but that the health system needed to ensure they were deployed equitably [45]. A potential policy direction could be to direct women away from the 26% of public clinics that have neither a midwife nor doctor on staff, and encourage them instead to give birth at facilities with sufficient staffing.

The low volumes of caesarean sections among adequately prepared facilities are another area of concern. There is a critical shortage of skilled surgical providers and that individual providers have low productivity, carrying out a median of just one surgery a week [46]. Given that population coverage of caesarean section is dangerously low, more investigation must be done to understand why these facilities- which can nominally provide caesarean sections- carry out so few, and if there are cost barriers or deficiencies in needed equipment, commodities or skills (e.g. anaesthetists). It might be necessary to provide more support in terms of staffing, training, supplies and infrastructure so that caesarean sections can be done whenever required in such facilities.

This study uses three good quality nationally representative data sources: DHS surveys using nationally representative samples, a census of all health facilities in Liberia (SARA 2018), and HMIS data including all health facilities in the country. The SARA 2018 census was comprehensive, with just 66 of the 831 (8%) facilities listed on the Ministry of Health register unreachable by the data collectors, perhaps representing non-operational facilities rather than missing data. A limitation of the datasets is that 80 of the 614 facilities (13%) in the SARA 2018 could not be matched to a corresponding entry from HMIS 2019. 84% of those were private clinics, 11% public clinics, 4% private health centres and 1% public health centres. 25 of the 586 (4%) facilities in HMIS could not be matched to the SARA, of which 84% were clinics and 16% health centres. While some of those facilities may have closed or opened in between the two timepoints, it is important to note that a substantial number of facilities in Liberia are excluded from this analysis, and volumes may be overestimated since private clinics were most likely to be missing.

The use of signal functions to assess emergency obstetric care also has limitations. It relies on self-report that an intervention has been performed in the last 12 months, and cannot assess whether the intervention was performed safely or correctly, or if staff were, or still are, appropriately qualified and trained to do so. This may lead to an overestimate of EmONC readiness. On the other hand, low volume facilities may not have experienced a less common obstetric complication (such as eclampsia) in the last 12 months, and could be recorded as not having the ability to carry out the signal function even if they could address the emergency in the rare circumstance of being presented with it.

## Conclusion

The Liberian health system has achieved a huge increase in facility births in recent years, mostly through women shifting from giving birth at home to low level public clinics. However, this study suggests that these women will not necessarily have safer births: most facilities are low volume and ill-prepared to deal with obstetric emergencies. To make more progress on reducing maternal and neonatal deaths, the Ministry of Health must shift from using percentage of births in a health facility as its main indicator to ensuring those facilities can offer safe care, of a much better quality than at home.

## Supplementary Information

**Additional file 1: Fig. S1.** Yearly volume of births in health facilities in Liberia (SARA 2018 and HMIS 2019). **Table S1.** Location of delivery indicators definitions. **Table S2.** EmONC signal functions definitions. **Table S3.** Place of delivery by midpoint of DHS recall period and location type. **Table S4.** Facilities in SARA 2018 offering delivery services. **Table S5.** Individual EmONC signal functions and EmONC classification by facility level and sector. **Table S6.** Individual EmONC signal functions and EmONC classification by facility volume. **Table S7.** Skilled birth attendants by cadre, SARA 2018.

## Data Availability

For DHS data, the datasets generated and/or analysed during the current study are available in the DHS Program repository, https://dhsprogram.com For SARA and HMIS data, the data that support the findings of this study are available from the Ministry of Health, Liberia but restrictions apply to the availability of these data, which were used under license for the current study, and so are not publicly available. Data are however available from the authors upon reasonable request (Patrick K Konwloh, pkkonwloh2006@gmail.com) and with permission of the Ministry of Health, Liberia.
