# Supplementary material for: Readiness of health facilities to provide safe childbirth in Liberia: a cross-sectional analysis of population surveys, facility censuses and facility birth records
Source: BMC Pregnancy Childbirth. 2022 Dec 20;22:952. doi: 10.1186/s12884-022-05301-x (PMC9764703; doi:10.1186/s12884-022-05301-x)
Supplement: Supplementary file 1 — Additional file 1: Fig. S1. Yearly volume of births in health facilities in Liberia (SARA 2018 and HMIS 2019). Table S1. Location of delivery indicators definitions. Table S2. EmONC signal functions definitions. Table S3. Place of delivery by midpoint of DHS recall period and location type. Table S4. Facilities in SARA 2018 offering delivery services. Table S5. Individual EmONC signal functions and EmONC classification by facility level and sector. Table S6. Individual EmONC signal functions and EmONC classification by facility volume. Table S7. Skilled birth attendants by cadre, SARA 2018. [file 12884_2022_5301_MOESM1_ESM.docx]

# Appendix

Figure A1: Yearly volume of births in health facilities in Liberia (SARA 2018 and HMIS 2019).

| Indicator | Responses included |
| --- | --- |
| Public hospital | Public hospital |
| Public health centre | Public health centre |
| Public clinic/other public | Public clinic; other public sector |
| Private | Private hospital/clinic; other private medical |
| Home | Respondent’s home; other home |
| Other/missing | Other; missing |
| Facility based delivery | Public hospital; Public health centre; Public clinic; other public sector; Private hospital/clinic; other private medical |

Table A1: Location of delivery indicators definitions

| Indicator | Self-report | Observation |
| --- | --- | --- |
| Parenteral antibiotics | Parenteral administration of antibiotics (IV or IM) for mothers carried out in the last 12 months | Non-expired injection or powder for injection of: gentamicin, ampicillin, metronidazole, benzathine benzylpenicillin, ceftriaxone |
| Parenteral oxytocin | Parenteral administration of oxytocin for treatment of post‐partum haemorrhage (IV or IM) carried out in last 12 months, or routine administration of oxytocin injection immediately after birth to all women for prevention of post-partum haemorrhage | Non-expired oxytocin injection |
| Parenteral anticonvulsant | Parenteral administration of magnesium sulphate for management of preeclampsia and eclampsia (IV or IM) carried out in last 12 months | Non-expired magnesium sulphate injectable |
| Manual removal placenta | Manual removal of placenta carried out in last 12 months | - |
| Removal retained products | One of the following carried out in last 12 months: removal of retained products of conception (MVA, vacuum aspiration), or dilation and curettage | Vacuum aspirator or D&C kit |
| Assisted vaginal delivery | Assisted vaginal delivery (vacuum extraction or forceps) carried out in last 12 months | Manual vacuum extractor (if only vacuum extraction assisted delivery reported) |
| Neonatal resuscitation | Neonatal resuscitation with bag and mask carried out in last 12 months | Newborn bag and mask size 0 or 1 |
| Caesarean section | Caesarean section carried out in last 12 months | - |
| Blood transfusion | Blood transfusion carried out in last 12 months | - |

Table A2: EmONC signal functions definitions

|  | National | | | Rural | | | | Urban | | | |
| --- | --- | --- | --- | --- | --- | --- | --- | --- | --- | --- | --- |
|  | 2004 | 2010 | 2017 | | 2004 | 2010 | 2017 | | 2004 | 2010 | 2017 |
| Home | 61.0  (57.2-64.8) | 43.6  (40.4-46.7) | 19.4  (16.8-22.0) | | 72.2  (67.4 - 77.1) | 53.5  (49.8 - 57.3) | 23.1  (19.8-26.4) | | 35.0  (24.0-30.1) | 33.6  (28.3-38.9) | 16.2  (12.3-20.2) |
| Public clinic/other public facility | 6.7  (4.8-8.6) | 17.4  15.2-19.7) | 34.1  (29.8-38.4) | | 6.1  (3.5-8.7) | 19.3  (16.4-22.2) | 48.3  (43.5-53.1) | | 8.1  (5.9-10.3) | 15.5  (12.1-19.0 | 21.7  (14.5-28.9) |
| Public health centre | 7.6  (5.7-9.4) | 3.1  (2.3-3.9) | 5.1  (3.7-6.4) | | 7.6  (5.2-10.0) | 3.8  (2.6-4.9) | 5.1  (3.9-6.3) | | 7.6  (5.1-10.2) | 2.5  (1.2-3.7) | 5.0  (2.8-7.3) |
| Public hospital | 12.7  (11.2-14.2) | 22.8  (20.2-25.4) | 26.3  (23.1-29.5) | | 6.5  (4.8-8.1) | 16.3  (13.4-19.1) | 16.3  (14.0-18.7) | | 27.1  (24.0-30.1) | 29.5  (24.8-34.1) | 35.1  (29.3-40.8) |
| Private facility (all levels) | 9.9  (8.4-11.5) | 12.5  (9.5-15.4) | 14.3  (11.5-17.2) | | 5.4  (3.7-7.0) | 6.2  (2.6-4.9) | 6.1  (4.4-7.7) | | 20.5  (17.8-23.2) | 18.7  (13.3-24.1) | 21.6  (16.5-26.7) |
| Other/missing | 2.1  (1.6-2.6) | 0.6  (0.3-0.9) | 0.7  (0.5-1.0) | | 2.3  (1.7-2.9) | 1.0  (0.2-1.5) | 1.1  (0.7-1.60) | | 1.7  (0.8-2.6) | 0.2  (0.0-0.4) | 0.4  (0.0-0.8) |
| **Overall FBD** | **36.9**  **(33.1-40.7)** | **55.8**  **(52.6-59.0)** | **79.8**  **(77.2-82.5)** | | **25.5**  **(20.6-30.3)** | **45.5**  **(41.7-49.4)** | **75.8**  **(72.4-79.2)** | | **63.2**  **(59.7-66.8)** | **66.2**  **(60.9-71.5)** | **83.4**  **(79.4-87.3)** |
| % of births via caesareean section | 3.3 (2.8 - 3.8) | 3.7 (3.0 - 4.3) | 5.0 (4.0- 6.0) | | 2.5 (1.9 - 3.1) | 2.5 (1.8- 3.3) | 3.7 (2.8 - 4.5) | | 5.1 (4.0 - 6.2) | 4.8 (3.6 - 6.0) | 6.1 (4.4 - 7.9) |
| *Carried out in public sector* | *2.0 (1.6-2.5)* | *2.8 (2.2- 3.4)* | *3.9 (3.1 - 4.7)* | | *1.7 (1.2 - 2.2)* | *1.9 (1.4 - 2.5)* | *3.1 (2.4 - 3.9)* | | *2.9 (2.0 - 2.8)* | *3.6 (2.5 - 4.6)* | *4.5 (3.2-5.9)* |
| *Carried out in private sector* | *1.3 (0.9-1.6)* | *0.9 (0.5 - 1.3)* | *1.1 (0.5 - 1.7)* | | *0.8 (0.4 - 1.2)* | *0.6 (0.2 - 1.0)* | *0.5 (0.2 - 0.8)* | | *2.2 (1.4 - 3.1)* | *1.2 (0.6 - 1.8)* | *1.6 (0.5 - 2.6)* |

Table A3: Place of delivery by midpoint of DHS recall period and location type.

|  | **Public hospitals** | **Public health centres** | **Public clinics** | **Private hospitals** | **Private health centres** | **Private clinics** | **Total** |
| --- | --- | --- | --- | --- | --- | --- | --- |
| **N** | 25 | 34 | 382 | 11 | 23 | 255 | **730** |
| **Offering delivery services** | 24  (96.0%) | 33  (97.1%) | 362  (94.8%) | 10  (90.9%) | 16  (69.6%) | 169  (66.3%) | **614**  **(84.1%)** |

Table A4: Facilities in SARA 2018 offering delivery services

|  | Public hospitals | Public health centres | Public clinics | Private hospitals | Private health centres | Private clinics | Total |
| --- | --- | --- | --- | --- | --- | --- | --- |
| Antibiotics | 100.0% | 100.0% | 97.5% | 100.0% | 100.0% | 92.3% | 96.4% |
| Manual removal placenta | 95.8% | 97.0% | 90.9% | 100.0% | 93.8% | 87.6% | 90.7% |
| Oxytocin | 95.8% | 93.9% | 92.5% | 100.0% | 93.8% | 81.1% | 89.7% |
| Neonatal resuscitation | 100.0% | 97.0% | 86.5% | 100.0% | 75.0% | 47.9% | 76.9% |
| Anticonvulsant | 87.5% | 84.9% | 63.5% | 100.0% | 62.5% | 41.4% | 60.1% |
| Removal retained products | 79.2% | 60.6% | 25.4% | 70.0% | 50.0% | 20.0% | 31.8% |
| Assisted vaginal delivery | 58.3% | 21.2% | 4.7% | 60.0% | 25.0% | 23.7% | 14.3% |
| Blood transfusion | 91.7% | 12.1% | 0.6% | 100.0% | 50.0% | 10.1% | 10.3% |
| Caesarean section | 91.7% | 6.1% | 1.1% | 100.0% | 43.8% | 6.5% | 9.1% |
| Median signal functions | 9 | 6 | 5 | 8.5 | 5.5 | 4 | 5 |
| Less than BEmONC-1 | 8.3% | 48.5% | 87.6% | 0.0% | 50.0% | 85.8% | 79.5% |
| BEmONC-1 | 0.0% | 45.5% | 12.4% | 0.0% | 18.8% | 9.5% | 12.9% |
| CEmONC but not BeMONC-1 | 20.8% | 0.0% | 0.0% | 30.0% | 6.3% | 2.4% | 2.1% |
| CEmONC -1 | 70.8% | 6.1% | 0.0% | 70.0% | 25.0% | 2.4% | 5.5% |
| Referral readiness (vehicle onsite or vehicle offsite + phone) | 91.7% | 68.7% | 32.9% | 100.0% | 75.0% | 43.8% | 42.4% |

Table A5: Individual EmONC signal functions and EmONC classification by facility level and sector

| Yearly volume of births | <52 (n=99) | 53-183 (n=231) | 184-365 (n=128) | 366-500 (n=37) | >500 (n=39) |
| --- | --- | --- | --- | --- | --- |
| Antibiotics | 94.9% | 96.5% | 98.4% | 100.0% | 100.0% |
| Manual removal placenta | 86.9% | 92.2% | 93.0% | 94.6% | 97.4% |
| Oxytocin | 85.9% | 90.0% | 93.0% | 100.0% | 97.4% |
| Neonatal resuscitation | 66.7% | 84.4% | 83.6% | 91.9% | 97.4% |
| Anticonvulsant | 45.5% | 63.2% | 64.8% | 78.4% | 89.7% |
| Removal retained products | 31.3% | 24.7% | 34.4% | 43.2% | 66.7% |
| Assisted vaginal delivery | 21.2% | 8.2% | 9.4% | 16.2% | 41.0% |
| Blood transfusion | 11.1% | 5.6% | 4.7% | 18.9% | 48.7% |
| Caesarean section | 9.1% | 3.9% | 6.3% | 18.9% | 46.2% |
| Median signal functions | 5 | 5 | 5 | 5 | 7 |
| Less than BEmONC-1 | 79.8% | 85.7% | 81.3% | 62.2% | 33.3% |
| CEmONC but not BeMONC-1 | 4.0% | 0.4% | 1.6% | 5.4% | 7.7% |
| BEmONC-1 | 12.1% | 12.1% | 14.8% | 18.9% | 20.5% |
| CEmONC -1 | 4.0% | 1.7% | 2.3% | 13.5% | 38.5% |
| Referral readiness (vehicle onsite or vehicle offsite + phone) | 45.5% | 36.8% | 43.0% | 29.7% | 74.4% |

Table A6: Individual EmONC signal functions and EmONC classification by facility volume

|  | Median employed by each facility (IQR) | | | | % employing at least one | | | |
| --- | --- | --- | --- | --- | --- | --- | --- | --- |
| Level and sector of facility | Doctor | Midwife | Nurse | **All SBA** | Doctor | Midwife | Nurse | **All SBA** |
| Public hospital N=23 | 7 (4-12) | 11 (3-16) | 26 (15-47) | **50 (28-75)** | 95.7 | 100.0 | 95.7 | **100.0** |
| Public health centre  N=33 | 1 (0-2) | 3 (2-7) | 6 (4-10) | **10 (7-16)** | 72.7 | 100.0 | 97.0 | **100.0** |
| Public clinic  N=362 | 0 (0-0) | 1 (0-1) | 2 (1-2) | **3 (2-3)** | 13.0 | 71.3 | 93.9 | **99.7** |
| Private hospital  N=10 | 4.5 (3-6) | 7 (5-10) | 23 (10-31) | **37 (20-45)** | 90.0 | 100.0 | 100.0 | **100.0** |
| Private health centre  N=15 | 2 (1-3) | 3 (1-11) | 4 (2-6) | **8 (6-13)** | 100.0 | 93.3 | 93.3 | **100.0** |
| Private clinic  N=169 | 1 (0-3) | 1 (1-2) | 2 (1-3) | **5(3-6)** | 71.6 | 86.4 | 92.9 | **99.4** |
| **All facilities**  **N=612** | **0 (0-1)** | **1 (1-2)** | **2 (1-3)** | **3 (2-6)** | **38.9** | **79.1** | **94.0** | **99.7** |

Table A7: Skilled birth attendants by cadre, SARA 2018
